# Indoxyl Sulfate, a Uremic Toxin, Stimulates Reactive Oxygen Species Production and Erythrocyte Cell Death Supposedly by an Organic Anion Transporter 2 (OAT2) and NADPH Oxidase Activity-Dependent Pathways

**DOI:** 10.3390/toxins10070280

**Published:** 2018-07-05

**Authors:** Gabriela Ferreira Dias, Natalia Borges Bonan, Thiago Maass Steiner, Sara Soares Tozoni, Silvia Rodrigues, Lia Sumie Nakao, Viktoriya Kuntsevich, Roberto Pecoits Filho, Peter Kotanko, Andréa N. Moreno-Amaral

**Affiliations:** 1 Graduate Program in Health Sciences, School of Medicine, Pontifícia Universidade Católica do Paraná; Curitiba-PR 80215-901, Brasilien; fdias.gabriela@gmail.com (G.F.D.); natybonan@globo.com (N.B.B.); thiago.m.steiner@gmail.com (T.M.S.); saratozoni@hotmail.com (S.S.T.); r.pecoits@pucpr.br (R.P.F.); 2 Department of Basic Pathology, Federal University of Paraná, Curitiba-PR 80060-000, Brazil; silviahellfire@gmail.com (S.R.); lia.nakao@ufpr.br (L.S.N.); 3 Renal Research Institute, New York, NY 10065, USA; vkshplsnchk@gmail.com (V.K.); Peter.Kotanko@rriny.com (P.K.)

**Keywords:** chronic kidney disease, indoxyl sulfate, eryptosis, oxidative stress

## Abstract

It is hypothesized that the uremic toxin indoxyl sulfate (IS) plays a role in the pathogenesis of renal anemia. To further explore that hypothesis, we examined the effects of IS on reactive oxygen species (ROS) production, levels of reduced glutathione (GSH), and erythrocyte death (eryptosis) in red blood cells (RBC) from healthy controls (CON-RBC) and hemodialyzed patients (HD-RBC), respectively. RBC were incubated either in either TRIS-Glc-BSA buffer or IS at concentrations of 0.01, 0.09, and 0.17 mM, respectively. We measured ROS generation (expressed as % of DCFH-DA positive RBC), eryptosis (expressed as % of annexin-V positive RBC), and GSH levels after 6, 12, and 24 h. When incubated in buffer, ROS production was approximately seven-fold higher at all time points HD-RBC when compared to CON-RBC. Incubation with IS increased ROS production in CON-RBS dose-dependently up to 10-fold. Eryptosis in buffer-incubated HD-RBC was up to seven-fold higher as compared to COB-RBC. Incubation of CON-RBC with IS increased the eryptosis rate dose-dependently up to 6-fold. Pretreatment of CON-RBC with the organic anion transporter 2 (OAT2) specific inhibitor ketoprofen or with NADPH oxidase inhibitor diphenyleneiodonium-Cl blunted the IS effect on both ROS production and eryptosis induction. While GSH levels in HD-RBC were reduced when compared to CON-RBC, they were not affected by IS incubation. In summary, IS increases ROS generation and eryptosis in CON-RBC by an activity dependent of the IS influx through OAT2, and NADPH oxidase activity-dependent, and a GSH-independent mechanism. These findings lend support to a putative role of IS in the pathogenesis of renal anemia.

## 1. Introduction

Anemia is a common complication in chronic kidney disease (CKD). Although renal anemia is predominantly caused by an impaired renal erythropoietin synthesis, it can be worsened by other factors [1]. Erythropoiesis stimulating agents (ESA) have a limited capacity of raising hemoglobin levels in inflamed patients, suggesting the contribution of inflammation in erythropoietin hyporesponsiveness, in which shortened red blood cell life span due to premature erythrocyte death (eryptosis) is also observed [2]. Many of these mechanisms are thought to induce oxidative stress or an altered ratio of pro-oxidative to antioxidant molecules within red blood cells (RBC). This imbalance can then result in the activation of Ca^2+^ and Cl^−^ channels, leading to cellular shrinkage and subsequent eryptosis [3].

Previous studies have shown that toxins, such as vanadate [4], acrolein [5], methylglyoxal [6], and indoxyl sulfate (IS) [7] cause eryptosis via not yet fully understood mechanisms. IS could well contribute to the accelerated erythrocyte death (eryptosis) in CKD [7] as well increase the risk of interference with microcirculation by PS exposure [8]. In addition, both free indoxyl sulfate (IS) and total IS, were significantly associated with erythropoietin levels [9] in CKD patients; this correlation was also demonstrated by IS suppressing erythropoietin (EPO) mRNA expression, via the disturbance in oxygen metabolism [10] and attenuating EPO-induced tyrosine phosphorylation of EPO receptor leading to an EPO resistance [11].

It is known that uremic toxins accumulate in various tissues, via organic anion transporters (OATs), causing damage. OAT1 and OAT3 have previously been shown to interact with many of uremic toxins in vitro and some in vivo data indicate that the OATs also handle many endogenous metabolites [12,13,14]. Recently, Sager and collaborators [15] characterized the transporters that are responsible for the uptake of cyclic nucleotides to human RBC surface and by western blotting, showing an expression of OAT2, but no expression of OAT1 or OAT3. Human-OAT2 was observed at the basolateral side of the proximal tubule, where it mediates the transport of organic anions, including salicylate and prostaglandin F2a, as well as interacting with IS [16,17]. However, the relationship between IS and OATs on erythrocytes has not been clarified. 

Under these circumstances the aim of the present study was to investigate mechanism by which the uremic toxin IS induces oxidative stress in erythrocytes and eryptosis. We incubated red blood cells (RBC) from healthy control (CON-RBC) with different concentrations of IS in the presence or absence of different inhibitors for the initial mechanism of IS influx or for oxidative stress pathway and measured (1) generation of reactive oxygen species (ROS); (2) eryptosis rate; and, (3) levels of reduced glutathione (GSH). All of these analyses were also made with RBC from hemodialysis patients (HD-RBC) and compared to CON-RBC.

## 2. Results

RBC were obtained from HD patients (HD-RBC) consisting of five male and five female patients with a mean age of 36.7 ± 4.5 years, and healthy subjects (CON-RBC) consisting of three male and five females with a mean age of 33.1 ± 11. All of the baseline characteristics of the patient population are presented in Table 1.

### 2.1. ROS Generation and Eryptosis

**Figure 1:**
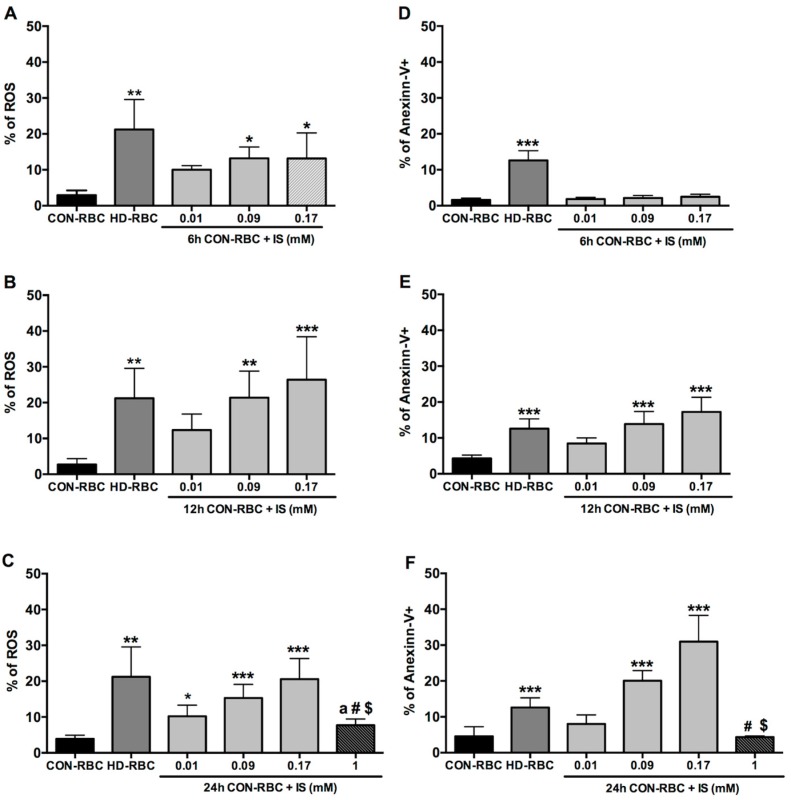
Indoxyl sulfate promotes reactive oxygen species (ROS) production and concurrent eryptosis in a time-dose response. Healthy controls (CON-RBC): healthy red blood cells incubated in TRIS-Glucose-BSA buffer, hemodialyzed patients (HD-RBC): freshly collected sample from hemodialysis patients (*n* = 10); not incubated, IS: indoxyl sulfate. CON-RBC + IS incubated in different concentrations of IS (0.01, 0.09, and 0.17 mM) for 6 h (*n* = 6) (**A**,**D**); 12 h (*n* = 8) (**B**,**E**); and 24 h (additional IS concentration 1 mM) (*n* = 8) (**C**,**F**). Levels of ROS production (**A**–**C**) are expressed as percentage of 2′,7′-dichlorodihydrofluorescein diacetate (DCFH-DA)-positive cells, eryptosis (**D**–**F**) expressed as percentage of annexin-V-positive cells. * *p* < 0.05; ** *p* < 0.01; *** *p* < 0.001 vs. CON-RBC incubated in TRIS-Glc-BSA buffer. **a** *p* < 0.001 vs. HD-RBC; **#** *p* < 0.05 vs. IS 0.09 mM; **$** *p* < 0.001 vs. IS 0.17 mM.

ROS generation in CON-RBC incubated with IS increased in a dose-dependent manner (Figure 1A–C). Of note, ROS production in freshly collected not incubated HD-RBC was with 21.2 ± 8.3% higher than in CON-RBC incubated in TRIS-Glc-BSA and comparable to levels that are seen in CON-RBC incubated for 12–24 h in 0.17 mM IS (Figure 1B,C, respectively).

While the eryptosis rate in CON-RBC that was incubated in TRIS-Glc-BSA tripled between 12 and 24 h, this increase was substantially more pronounced in IS-incubated CON-RBC, where eryptosis increased more than 10-fold at the highest IS level (Figure 1E,F, respectively). The eryptosis rate in freshly collected HD-RBC was with 12.5 ± 2.7%, which is similar to one observed in CON-RBC after 12 h incubation in IS at 0.09 mM and 0.17 mM, respectively (Figure 1E). 

Interestingly, both ROS generation and eryptosis were inhibited after 24 h of incubation with IS used in higher concentration (1 mM), (Figure 1C,F, respectively), as a negative feedback by the transporter of the IS influx into the cells.

**Figure 2:**
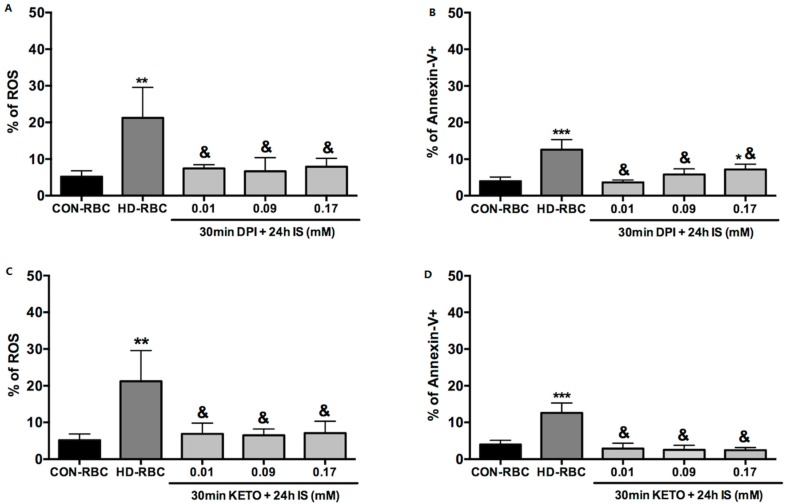
ROS production and eryptosis induced by Indoxyl Sulfate were abolished in the presence of diphenyleneiodonium chloride (DPI), an inhibitor of NADPH oxidase, and also in the presence of ketoprofen (KETO) an inhibitor of Organic Anion Transporter 2 (OAT2). CON-RBC: healthy red blood cells incubated in TRIS-Glucose-BSA buffer (baseline), HD-RBC: freshly collected sample from hemodialysis patients. CON-RBC were pretreated with DPI (10 µM) (**A**,**B**) or KETO (30 mM) (**C**,**D**) for 30 min before 24-h incubation in different concentrations of IS (0.01, 0.09, and 0.17 mM). Levels of ROS production (**A** and **C**) are expressed as percentage of 2′,7′-dichlorodihydrofluorescein diacetate (DCFH-DA)-positive cells, eryptosis (**B** and **D**) expressed as percentage of annexin-V-positive cells. *n* = 6 for ROS, *n* = 6 for eryptosis. * *p* < 0.05; ** *p* < 0.001; *** *p* < 0.0001 vs. CON-RBC baseline; **&** *p* < 0.001 vs. HD-RBC.

Thirty minutes of pre-treatment of CON-RBC with diphenyleneiodonium chloride (DPI) (10 µM), which is an inhibitor of NADPH oxidase, or with ketoprofen (KETO) (30 μM) an OAT2 specific inhibitor, blunted the effect of a 24-h incubation in IS on both ROS production and eryptosis (Figure 2).

### 2.2. Glutathione Levels

**Figure 3:**
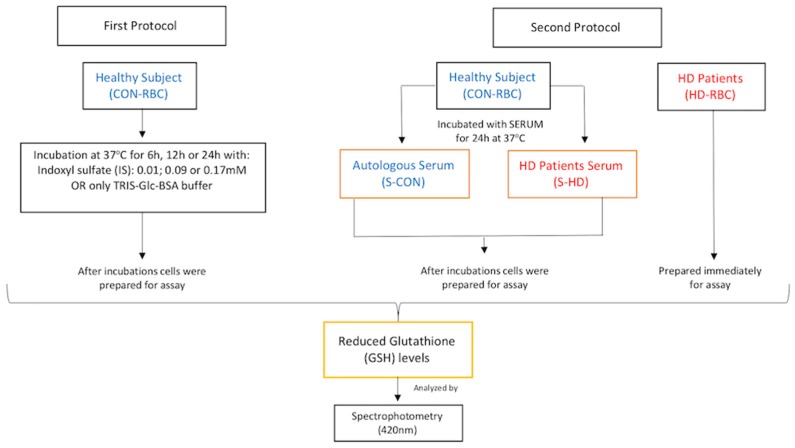
Schematic view of the first and second protocol for obtaining, preparing and incubating the cells GHS analysis.

We measured GSH levels in (1) CON-RBC incubated in different IS concentrations (Figure 3—First Protocol); (2) CON-RBC incubated in autologous serum or in serum obtained from HD-patient; and, (3) Freshly collected not incubated HD-RBC from hemodialysis patients (Figure 3—Second Protocol). As a positive control of GSH levels, CON-RBC were also incubated in tert-Butyl hydroperoxide (TBHP, 5 mM), which is a substance that lowers intracellular GSH levels.

**Figure 4:**
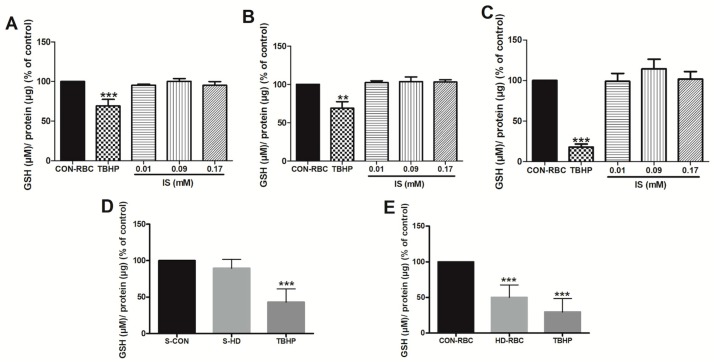
Effect of indoxyl sulfate on GSH levels. GSH: reduced glutathione, CON-RBC/TRIS-Glc: healthy red blood cells incubated in Tris-Glucose-BSA buffer only, TBHP: CON-RBC incubated in tert-Butyl hydroperoxide, IS: CON-RBC incubated in indoxyl sulfate, CON-RBC vs. S-CON: heathy red blood cells incubated in autologous serum, CON-RBC vs. S-HD: CON-RBC incubated in serum obtained from hemodialysis patients, CON-RBC: healthy erythrocytes, HD-RBC: hemodialysis patient erythrocyte. Top row (Panels **A**–**C**) show values at three different incubation times (**A**) = 6 h; (**B**) = 12 h; (**C**) = 24 h. In the bottom row (Panels **D**,**E**), incubation times were 24 h. Panel (**D**) shows CON-RBC exposed to autologous serum (S-CON) or serum from dialysis patients (S-HD). Panel (**E**) shows results in CON-RBC vs. HD-RBC. Data are expressed as percentage of the control, which was considered to be 100%. ** *p* < 0.001; *** *p* < 0.0001.

Results are shown in Figure 4. The top row shows that in CON-RBC incubated with different IS concentrations did not have a noticeable effect on GSH levels after 6, 12, and 24 h incubation (Panels A–C, respectively), while incubation in the positive control TBHP caused a substantial GSH decrease. 

The bottom row of Figure 4, Panel D shows that both autologous serum (S-CON) or dialysis patient serum (S-HD) had no effect on the reduction of GSH levels at 24 h in CON-RBC, but the same cells that were incubated with positive control TBHP showed the expected GSH decline. Panel E shows baseline levels of GSH in CON-RBC vs. HD-RBC. GSH levels decline by ~50% in HD-RBC. Incubation in TBHP of the CON-RBC markedly lowered the GSH levels.

## 3. Discussion

Previous research has found that IS can promote elevated eryptosis in-vitro [8], and this process can be linked to the presence of ROS in cells [18,19]. However, the overall mechanism that is responsible for these effects are not fully understood, and in particular, there is little information regarding the pathway of IS to cause the RBC damage. In this study, we identified, for the first time, organic anion transporter 2 (OAT2) as important contributor to IS-induced RBC death in a dependent NADPH oxidase activity in a GSH-independent mechanism.

Uremic toxicity impacts cells and tissues either by direct harm in the cell surface or through in-cell toxicity when internalization occurs. Here, we describe that the IS effects on ROS production and PS exposure by erythrocytes were abrogated by the presence of a specific inhibitor for organic anion transporter 2 (OAT2), suggesting internalization as a mechanism for IS uremic toxicity to RBCs. Sager et al. [15] described not only the OAT2 expression on RBC surface, but also its activity with the uptake of organic anions, such as cyclic nucleotides, creatinine, and indomethacin. OAT2 is a member of the SLC family (SLC22) that mediates the uptake of organic ions and it is associated to renal clearance of several drugs [20,21,22,23]. To the best of our knowledge, this is the first time that a mechanism by which IS influx through OAT2 causes severe damage in RBC is investigated. In the presence of ketoprofen, which is a specific inhibitor of OAT2, all of actions that we described for IS on human RBC were completely abrogated. The present study suggests that the OAT2 present in the RBC surface (allowing for the IS internalization in the erythrocyte) plays an essential process, the eryptosis process. 

Another potential mechanism of IS-induced eryptosis, as described in the present study, involves the NADPH oxidase-dependent pathway, since the eryptotic and oxidative stress effects of IS were inhibited by DPI. A relationship between the IS effect on ROS generation was suggested in studies that are describing that the effects of ROS were blocked by preincubating target-cells in DPI [24,25]. NADPH oxidase family enzymes (or NOXs) are unique in ROS production as their primary function and appear to be especially well suited for involvement in redox signaling [26]. Studying the oxidative stress in sickle cell disease, George and collaborators demonstrated the presence not only of NOX1 and NOX5, but also catalytic subunits that are necessary to the NADPH oxidase complex in both normal and sickle erythrocytes, suggesting that these may be the primary active isoforms of NADPH oxidase-mediated ROS generation in erythrocytes [27]. The potential role of this mechanistic pathway in the erythrocyte response to uremic toxins has not been described until the present. However, some studies described IS as an inductor of ROS generation by upregulating Nox4 in human aortic smooth muscle cells, followed by aortic calcification [28] or in 3T3-L1 adipose cells increased ROS production mainly through activation of NADPH oxidase [29].

In addition, the results of the present study show higher ROS generation in HD-RBC when compared to CON-RBC, and eryptosis rates were markedly higher in HD-RBC vs. CON-RBC, findings that are supported by previously studies [8,30,31]. We found that when CON-RBC were exposed to increased concentrations of IS, both the level of ROS and the eryptosis rate increased in a dose-dependent manner. Nevertheless, we are cautious to ascribe the elevated levels of ROS and eryptosis in uremic erythrocytes just to the presence of IS. This characteristic of IS as a ROS-inducer has been shown in different cell types with adverse consequences, such as not only impair renal cells functions, but also aggravate the progression of CKD [18,19,32], and also has harmful effects on endothelial cells, since the ROS production induced by IS was observed the increase of the NADPH oxidase activity with decreased glutathione levels [24].

In healthy erythrocytes, 98% of glutathione is present in its active reduced form, GSH [33]. As a cell undergoes oxidative stress, the fraction of GSH decreases (and the fraction of the oxidized form GSSG increases), resulting in the diminished ability of the cell to neutralize ROS. Khazim et al., found that the GSH/GSSG redox potential was lower in erythrocytes that were obtained from hemodialysis patients than in those from healthy controls [34]. In addition, serum from hemodialysis patients decreased GSH, ascorbic acid [35], and diminished the activity of the antioxidant enzymes superoxide dismutase and GSH peroxidase [36]. In the present study, the results were largely negative with respect to IS. Even after prolonged incubation for up to 24 h and even at the highest concentration of IS tested (0.17 mM), we noted no GSH reduction in healthy erythrocytes. On the other hand, HD-RBC had a mere 50% of the GSH content that was found in CON-RBC. Incubation of CON-RBC in serum from dialysis patients had no effect on GSH levels, suggesting that this decrease was not related to some plasma inhibitor(s). Thus, our results suggest that the decreased GSH levels in erythrocytes from dialysis patients cannot be explained by exposure of these cells to IS. 

The clinical translation of the suggested involvement of uremic toxicity in renal anemia is still controversial. Wu et al., reported that total IS concentration was significantly associated with EPO levels in a cohort of one hundred and thirteen CKD stable patients [9]. In contrast, in an observational multicentric study, including 240 hemodialysis patients, no link between uremic toxins (Indole 3-acetic acid, indoxyl sulfate, and *p*-Cresyl-sulfate) and anemia was observed [37]. Therefore, it is clear that studies in larger cohorts of patients with CKD are important to investigate whether or not uremic toxins are important players in renal anemia, and whether this would offer novel opportunities for interventions in renal anemia that are focusing on different mechanisms.

Our study presents some limitations that need to be highlighted. First, we did not measure serum IS in the HD patients who donated the blood specimens. Second, we did not determine the intracellular IS after RBC incubation. Finally, we did not test other inhibitors of the oxidative stress nor eryptosis, and therefore further studies will need to investigate other specific pathways that are induced by IS. On the other hand, this was the first study to identify basic cellular mechanisms that are activated by the uremic toxin IS in the induction of eryptosis, with possible consequences in the development of renal anemia.

In conclusion, IS increases ROS generation and eryptosis in CON-RBC by an activity dependent of the IS influx through OAT2, and NADPH oxidase activity-dependent, and a GSH-independent mechanism. These findings lend support to a putative role of IS in the pathogenesis of renal anemia. Besides that, the IS actions on RBC regulated by OAT2 that are described in the present study could serve as an important target to decrease the adverse effects of uremic toxins in this specific cell type.

## 4. Materials and Methods

### 4.1. Subjects

This study was approved by the Ethics Committee of Pontifícia Universidade Católica do Paraná (PUCPR). Informed consent was obtained from all individuals (Approved on August 29, 2016 under registration number 1.752.213). Clinically stable HD patients (*n* = 10), in a thrice-weekly hemodialysis program for at least three months using high-flux dialyzers, were recruited from the dialysis unit of PUCPR. Criteria of inclusion form both HD patients and Healthy subjects were age >18 years old, provided written, informed consent prior to blood collection. Criteria of exclusion were the presence of acute or chronic infection, active immunological disease, and blood transfusion with-in the past month. Healthy subjects, defined after clinical interview were recruited from laboratory staff. Heparinized blood (5–10 mL) was obtained from healthy controls (*n* = 8) and patients (*n* = 10) on hemodialysis.

### 4.2. Blood Samples and Red Blood Cells (RBCs) Preparations and Incubations

**Figure 5:**
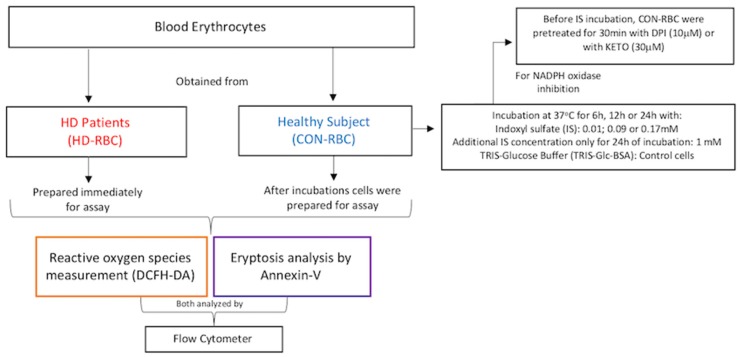
Schematic view of the protocol for obtaining, preparing and incubating the cells for ROS or eryptosis production analysis.

Blood samples were centrifuged at 3000 rpm for 15 min at room temperature. The buffy coat was carefully discarded, and the remaining erythrocytes were washed three times with cold phosphate buffered saline (PBS). The cells were then incubated for 6, 12, or 24 h at 37 °C at 0.5% hematocrit with 5% CO_2_ in different concentrations of indoxyl sulfate (Sigma-Aldrich, St. Louis, MO, USA) (IS—0.01 mM, 0.09 mM, and 0.17 mM, concentrations established by Duranton and coworkers [38]), diluted in Tris-Glucose-BSA buffer (mM—21.0 tris(hydroxymethyl)aminomethane; 4.7 KCl; 2.0 CaCl_2_, 140.5 NaCl; 1.2 MgSO_4_, and 5.5 glucose, added 4% of Bovine Serum Albumin (Sigma-Aldrich, St. Louis, MO, USA)—pH 7.4). In order to better understand whether the influx of the toxin would be a pathway for the erythrocyte activation, we used 1 mM of IS to incubate the RBCs for 24 h, concentration described by promote negative feedback in organic anion transporters (OAT) [17]. Erythrocytes that were subjected to inhibition of NADPH oxidase or Organic Anion Transport 2 (OAT2) were pretreated with diphenyleneiodonium (DPI—10 µM) or with ketoprofen (KETO) (30 μM) [39], respectively, for 30 min before the addition of indoxyl sulfate for 24 hours incubation. Additionally, higher IS concentration (1mM) was used as a positive saturable uptake [17]. For reduced glutathione dosage, 150 µL of pure RBC were used. Control erythrocytes were incubated for the same period of time in Tris-Glc-BSA buffer only (Figure 5).

### 4.3. Measurement of Reactive Oxygen Species Production

Following incubations, CON-RBC were centrifuged and resuspended in 2 mM of 2′,7′-dichlorodihydrofluorescein diacetate (DCFH-DA) (Abcam, Cambridge, MA, USA) for 30 min in the dark. The cells were then washed once with PBS and resuspended in 200 µL of PBS 0.4% formaldehyde (Fix-FACS). Reactive oxygen species fluorescence intensity was quantified by flow cytometry (FACS Calibur BD Bioscience, Sparks, MD, USA). Fresh HD-RBC were stained using the same technique (See Figure 4).

### 4.4. Measurement of Eryptosis Levels by Annexin-V Binding 

CON-RBC were stained with the cellular protein annexin-V-PE (BD Bioscience, Sparks, MD, USA) for 15 min in the dark, according the manufacturer’s instructions, washed once with phosphate buffer solution, and then resuspended in 200 µL of Fix-FACS. Annexin-V fluorescence intensity was analyzed by flow cytometry (FACS Calibur BD Bioscience, Sparks, MD, USA). HD-RBC were stained using the same technique (See Figure 4).

### 4.5. Measurement of Reduced Glutathione (GSH) Levels

First Protocol—After incubation in different concentrations of indoxyl sulfate for 6, 12, or 24 h, the CON-RBC suspension (50% hematocrit) were prepared to GSH analysis (Figure 2). 

Second Protocol—CON-RBC were also used to serum tests. Cells were incubated for 24 h in autologous serum (S-CON) or serum obtained from hemodialysis patients (S-HD). HD-RBC were collected and immediately prepared for GSH measurement (Figure 2). 

Positive control in both protocols—CON-RBC were incubated with 5 mM tert-Butyl hydroperoxide (TBHP) for 30 min in Tris-Glc-BSA buffer. 

After the incubations that were described above, erythrocytes (50% hematocrit) were deproteinized with 650 µL of solution containing 2 M perchloric acid and 4 mM diethylenetriaminepentaacetic acid (DTPA), and then centrifuged at 13,000 rpm for 10 min at 4 °C. On ice and under the protection from light, the pH of the supernatant (100 µL) was adjusted to ~7 with 800 µL of 300 mM phosphate buffer containing 10 mM ethylenediaminetetraacetic acid (EDTA). After a short spin in the centrifuge, the supernatant was incubated in a solution containing 0.25 mM Ellman’s Reagent (DTNB) in 300 mM phosphate buffer with 0.1 mM DTPA, pH 7.5. After 10 min of incubation, the absorbance of the sample was measured in a U-2900 Hitachi spectrophotometer (Hitachi High-Technologies Corporation, Tokyo, Japan) at 412 nm Protein content was determined from the initial 50% hematocrit suspension by the Bradford method.

### 4.6. Statistical Analysis 

All data are presented as mean ± SD or percentage as compared to the control, which was considered to be 100%. The significance of differences was determined by one-way analysis of variance (ANOVA), followed by a post hoc Tukey multiple comparisons test. All statistical analysis was performed using GraphPad Prism 5 software (GraphPad Software, La Jolla, CA, USA). *p* < 0.05 was considered to be statistically significant.

## Figures and Tables

**Table 1:** Clinical and Laboratory characteristics of the study Population.

Parameters	Healthy Subjects [*n* = 8]	HD Patients [*n* = 10]
Age (years)	33.1 ± 11	36.7 ± 4.5
Gender (Male %)	44.4	50
Caucasians (%)	100	93.3
BMI (Kg/m²)	22 ± 1.8	25.3 ± 3.7
Hb (g/dL)	14.1 ± 0.5	10.8 ± 1.2
Creatinine (mg/dL)	0.9 ± 2	12.4 ± 2.1
Urea normal or pre-HD (mg/dL)	21 ± 0.5	164.4 ± 41
Urea post HD (mg/dL)	NA	54.1 ± 17.7
CVD	0	21.1
DM	0	33.3
Hypertension	0	46.6

Data expressed as mean ± SD, or binary variables (frequency). BMI = Body mass index; Hb = hemoglobin; HD = hemodialysis; CVD = cardiovascular disease; DM = diabetes mellitus; NA = not available.
